# Carrier‐Free Self‐Assembly Nano‐Sonosensitizers for Sonodynamic‐Amplified Cuproptosis‐Ferroptosis in Glioblastoma Therapy

**DOI:** 10.1002/advs.202402516

**Published:** 2024-04-06

**Authors:** Yang Zhu, Xuegang Niu, Chengyu Ding, Yuanxiang Lin, Wenhua Fang, Lingjun Yan, Junjie Cheng, Jianhua Zou, Yu Tian, Wei Huang, Wen Huang, Yuanbo Pan, Tiantian Wu, Xiaoyuan Chen, Dezhi Kang

**Affiliations:** ^1^ Department of Neurosurgery Neurosurgery Research Institute The First Affiliated Hospital Fujian Medical University Fuzhou Fujian 350209 China; ^2^ Fujian Provincial Institutes of Brain Disorders and Brain Sciences The First Affiliated Hospital Fujian Medical University Fuzhou Fujian 350209 China; ^3^ Department of Neurosurgery National Regional Medical Center The First Affiliated Hospital Binhai Campus of the First Affiliated Hospital Fujian Medical University Fuzhou Fujian 350212 China; ^4^ Departments of Diagnostic Radiology, Surgery Chemical and Biomolecular Engineering, and Biomedical Engineering Yong Loo Lin School of Medicine and College of Design and Engineering National University of Singapore Singapore 119074 Singapore; ^5^ Clinical Imaging Research Centre Centre for Translational Medicine Yong Loo Lin School of Medicine National University of Singapore Singapore 117599 Singapore; ^6^ Nanomedicine Translational Research Program Yong Loo Lin School of Medicine National University of Singapore Singapore 117597 Singapore; ^7^ Institute of Molecular and Cell Biology Agency for Science Technology and Research (A*STAR) Proteos 61 Biopolis Drive Singapore 138673 Singapore; ^8^ School of Pharmaceutical Sciences/NHC key laboratory of tropical disease control/School of Tropical Medicine Hainan Medical University Haikou 571199 P. R. China

**Keywords:** carrier‐free nanoparticles, cuproptosis, ferroptosis, lipid peroxidation, sonodynamic therapy

## Abstract

Cuproptosis is a newly discovered form of programmed cell death significantly depending on the transport efficacy of copper (Cu) ionophores. However, existing Cu ionophores, primarily small molecules with a short blood half‐life, face challenges in transporting enough amounts of Cu ions into tumor cells. This work describes the construction of carrier‐free nanoparticles (Ce6@Cu NPs), which self‐assembled by the coordination of Cu^2+^ with the sonosensitizer chlorin e6 (Ce6), facilitating sonodynamic‐triggered combination of cuproptosis and ferroptosis. Ce6@Cu NPs internalized by U87MG cells induce a sonodynamic effect and glutathione (GSH) depletion capability, promoting lipid peroxidation and eventually inducing ferroptosis. Furthermore, Cu^+^ concentration in tumor cells significantly increases as Cu^2+^ reacts with reductive GSH, resulting in the downregulation of ferredoxin‐1 and lipoyl synthase. This induces the oligomerization of lipoylated dihydrolipoamide S‐acetyltransferase, causing proteotoxic stress and irreversible cuproptosis. Ce6@Cu NPs possess a satisfactory ability to penetrate the blood‐brain barrier, resulting in significant accumulation in orthotopic U87MG‐Luc glioblastoma. The sonodynamic‐triggered combination of ferroptosis and cuproptosis in the tumor by Ce6@Cu NPs is evidenced both in vitro and in vivo with minimal side effects. This work represents a promising tumor therapeutic strategy combining ferroptosis and cuproptosis, potentially inspiring further research in developing logical and effective cancer therapies based on cuproptosis.

## Graphical abstract

The carrier‐free self‐assembled Ce6@Cu nanoparticles are fabricated for sonodynamic‐therapy involving cuproptosis and ferroptosis. Ce6@Cu NPs show sonodynamic effect and glutathione depletion capability, promoting ferroptosis. Furthermore, the elevated copper concentration induces the downregulation of ferredoxin‐1, lipoyl synthase, and the oligomerization of lipoylated dihydrolipoamide S‐acetyltransferase, causing proteotoxic stress, and cuproptosis.

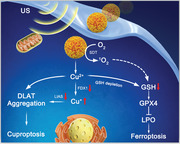

## 1 Introduction

Copper (Cu) is an intracellular trace metal that serves as an indispensable redox cofactor, involved in different cellular metabolic processes.^[^ ^1^, ^2^, ^3^ ^]^ A unique Cu‐dependent death pathway has been recently identified, which was called Cuproptosis.^[^ ^4^, ^5^, ^6^, ^7^, ^8^, ^9^, ^10^, ^11^, ^12^, ^13^, ^14^ ^]^ Cuproptosis is a new programmed cell death induced by Cu‐dependent mitochondrial dysfunction, which is distinct from apoptosis, necroptosis, ferroptosis, and pyroptosis.^[^ ^15^, ^16^, ^17^, ^18^, ^19^, ^20^ ^]^ Cuproptosis primarily relies on the accumulation of intracellular Cu into mitochondria, regulating tricarboxylic acid cycle (TCA) related metabolism. As regards the mechanism of action, ferredoxin‐1 (FDX1) and lipoyl synthase (LIAS) regulate dihydrolipoamide S‐acetyltransferase (DLAT) to undergo lipoylation.^[^ ^21^, ^22^, ^23^ ^]^ This results in the reduction of Cu^2+^ and the oligomerization of lipoylated DLAT, leading to proteotoxic stress and eventually cell death. In addition, the endogenous overproduced glutathione (GSH) acts as a natural Cu chaperone in cells inhibiting cuproptosis by working as a thiol‐containing copper chelator.^[^ ^24^, ^25^ ^]^ However, cuproptosis strongly depends on the transport efficiency of Cu ionophores.^[^ ^26^ ^]^ The unique transport and balancing mechanisms of Cu lead to a cellular Cu concentration that is not enough to induce cuproptosis.^[^ ^27^, ^28^ ^]^ Despites elesclomol is an effective Cu ionophore, it is inappropriate for clinical application due to its poor biostability and undesirable side effects.^[^ ^18^ ^]^ Thus, a safer cuproptosis inducer is needed for cancer therapy by effectively depleting GSH and delivering a sufficient amount of Cu ions.

Sonodynamic therapy (SDT) is a promising noninvasive cancer treatment due to its superior advantages in deeper tissue penetration and superior spatial precision.^[^ ^29^, ^30^, ^31^, ^32^, ^33^, ^34^, ^35^ ^]^ SDT activates sonosensitizer by ultrasound (US), producing plentiful reactive oxygen species (ROS) that induce oxidative stress, ultimately leading to cancer cell death.^[^ ^36^, ^37^, ^38^, ^39^, ^40^ ^]^ US‐triggered SDT possesses superior tissue penetration ability compared to light‐mediated therapeutic modalities like photothermal therapy and photodynamic therapy, thus offering a promising alternative for the controlled, noninvasive treatment of deep‐seated tumors like glioblastoma.^[^ ^41^, ^42^, ^43^ ^]^ In addition, the exogenous ROS generated by nano‐sonosensitizer during SDT can lead to the excessive accumulation of lipid peroxides, leading to irreversible ferroptosis.^[^ ^44^, ^45^, ^46^ ^]^ However, the elevated level of reductive GSH in tumor cells can scavenge excessive ROS generated by SDT, resulting in an ineffective inhibition of tumor growth.^[^ ^47^, ^48^ ^]^ Furthermore, the depletion of GSH can induce the inactivation of glutathione peroxides 4 (GPX4) and accelerate lipid peroxidation (LPO), triggering considerable ferroptosis.^[^ ^49^, ^50^, ^51^ ^]^ Therefore, it is crucial to develop nano‐sonosensitizers with the ability of synergistic GSH depletion and ROS generation to induce ferroptosis and perform an effective tumor therapy.

**Scheme 1:**
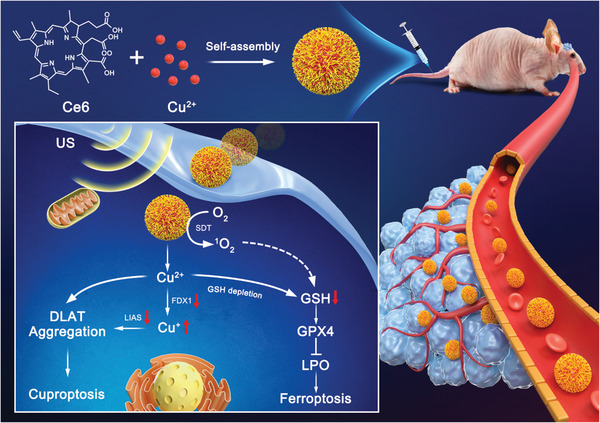
Schematic illustration of the fabrication of the carrier‐free Ce6@Cu NPs and the sonodynamic‐intensified cuproptosis‐ferroptosis in orthotopic U87MG‐Luc tumor therapy.

This work describes the construction of carrier‐free nanoparticles (Ce6@Cu NPs) that self‐assembled through the coordination of Cu^2+^ ions with sonosensitizer chlorin e6 (Ce6). This resulted in the induction of a sonodynamic effect, facilitating combination of ferroptosis and cuproptosis (**Scheme** **1**). Ce6@Cu NPs internalized into U87MG cells demonstrated and exerted an outstanding sonodynamic efficacy, producing abundant singlet oxygen (^1^O_2_) under US irradiation. This led to the oxidation of polyunsaturated fatty acids (PUFA), subsequently initiating lethal LPO. Furthermore, Ce6@Cu NPs also effectively depleted the overproduced reductive GSH in tumor cells, leading to the inactivation of GPX4, the acceleration of LPO, and the induction of irreversible ferroptosis. Besides, the Cu^+^ concentration in U87MG cells considerably elevated due to the reaction of Cu^2+^ with reductive GSH, resulting in the downregulation of FDX1 and LIAS expressions. This process significantly triggered the oligomerization of lipoylated DLAT, leading to proteotoxic stress and eventually inducing cell cuproptosis. Importantly, Ce6@Cu NPs showed a satisfactory ability to penetrate the blood‐brain barrier (BBB), leading to the prominent accumulation into orthotopic U87MG‐Luc glioblastoma. The sonodynamic‐triggered combination of ferroptosis and cuproptosis induced by Ce6@Cu NPs was demonstrated both in vitro and in vivo with minimal side effects. This work not only serves as a proof‐of‐concept for the fabrication of a carrier‐free nano‐sonosensitizer but also offers a promising tumor therapeutic strategy based on the synergistic ferroptosis and cuproptosis.

## 2 Results and Discussion

**Figure 1:**
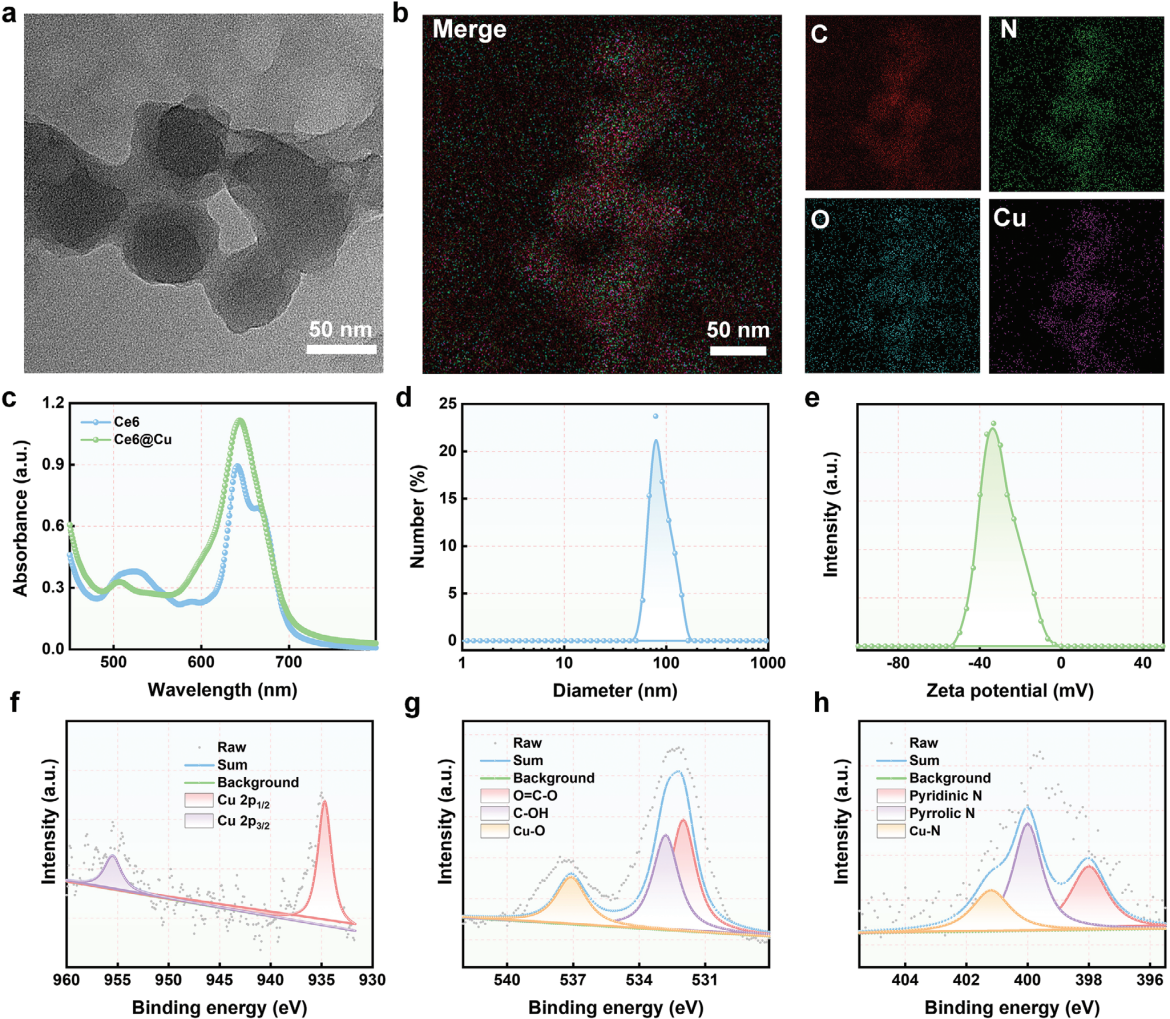
Characterization of Ce6@Cu NPs. a) TEM image of Ce6@Cu NPs. b) EDS mapping of Ce6@Cu NPs. c) UV–vis spectra of Ce6 and Ce6@Cu NPs. d) Diameter and e) zeta potential of Ce6@Cu NPs. f) High‐resolution XPS spectra of Cu 2p, g) O 1s, and h) N 1s in Ce6@Cu NPs.

The fabrication procedure is shown in Scheme 1. Carrier‐free Ce6@Cu NPs were constructed using a self‐assembly method. Spherical Ce6@Cu NPs, with a particle size of ≈50 nm were obtained as revealed by using transmission electron microscopy (TEM) (**Figure** **1**a). The corresponding energy‐dispersive X‐ray spectroscopy (EDS) mapping images demonstrated the distribution of Cu, carbon (C), notrogen (N), and oxygen (O) atoms in Ce6@Cu NPs (Figure 1b). The characteristic ultraviolet visible (UV–vis) absorption peak of Ce6 in Ce6@Cu NPs demonstrated the successful self‐assembly of Ce6 and Cu (Figure 1c). Furthermore, Fourier transform infrared spectra demonstrated that Ce6 and Ce6@Cu NPs had similar peaks (Figure S1, Supporting Information), further indicating the successful construction of Ce6@Cu NPs. The molar ratio between Cu and to Ce6 in Ce6@Cu NPs was ≈2.5:1, as determined using inductively coupled plasma mass spectrometry (ICP‐MS) and UV–vis spectrometry. Then, the hydrodynamic size and zeta potential of Ce6@Cu NPs were ≈80 nm and −33 mV, respectively, as measured by dynamic light scattering (DLS) (Figure 1d). No apparent crystal peak was noticed in the X‐ray powder diffraction (XRD) spectrum, implying the poor crystallinity of Cu element in Ce6@Cu NPs (Figure S2, Supporting Information). The X‐ray photoelectron spectroscopy (XPS) used to assess the Cu valences in the sample revealed that the high‐resolution Cu 2p XPS spectrum possessed two characteristic Cu^2+^ 2p peaks at Cu 2p_2/3_ (934.7 eV) and Cu 2p_1/3_ 955.6 eV (Figure 1g; Figure S3a,b, Supporting Information), implying the excellent GSH depletion capability of Ce6@Cu NPs. In addition, CuLM2 XPS further indicated Cu valence in Ce6@Cu NPs was +2 (Figure S3c, Supporting Information). The high‐resolution XPS spectra of O 1 and N 1 s clearly showed the coordination of Cu^2+^ with N and O atoms in the fabrication process of Ce6@Cu NPs (Figure 1g,h). In addition, the long‐term measurement of the stability studied by DLS demonstrated that the DLS result showed that the diameter of Ce6@Cu NPs remained stable for 120 h, suggesting the stability of Ce6@Cu NPs in a biological environment (Figure S4, Supporting Information). The drug release profile of Ce6@Cu NPs in various media examined through UV–vis spectroscopy (Figure S5, Supporting Information) revealed that Ce6@Cu NPs efficiently released Ce6 in the presence of 10 mM GSH. These findings verified the successful fabrication of Ce6@Cu NPs using a self‐assembly strategy.

**Figure 2:**
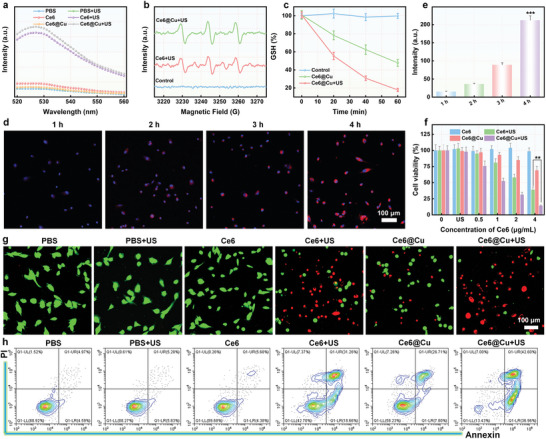
SDT performance and cytotoxicity of Ce6@Cu NPs. a) Fluorescence spectra of DCFH‐DA treated with Ce6@Cu NPs upon US irradiation. b) ESR spectra of Ce6@Cu NPs plus US irradiation using TEMP as the spin trapper. c) UV–vis spectra of DTNB treated with Ce6@Cu NPs upon US irradiation. d) CLSM images of Ce6@Cu NPs in U87MG cells and e) the corresponding quantification of fluorescence signals. f) Cell viability of tumor cells after treatment with Ce6@Cu NPs and Ce6@Cu NPs when exposed to US irradiation for 24 h. g) CLSM images demonstrated calcein‐AM/PI co‐staining of U87MG cells after 24 h incubation with various formulations. h) Determination of U87MG cell death via flow cytometry after the treatments with different formulations for 24 h. *n* = 5, P‐values are calculated using the Student's two‐tailed *t*‐test, ^**^ *p* < 0.05, ^***^ *p* < 0.001.

The sonodynamic effect of Ce6@Cu NPs measured using the fluorescence probe (2’,7’‐dichlorofluorescin diacetate, DCFH‐DA) showed that Ce6@Cu NPs induced a great increase in the fluorescence signal at 530 nm after US irradiation, suggesting the effective ROS generation (**Figure** **2**a; Figure S6, Supporting Information). Additionally, the specific fluorescence probe singlet oxygen sensor green (SOSG) was selected to detect ^1^O_2_ production in vitro and the results indicated that both Ce6@Cu NPs and free Ce6 induced a great increase in the fluorescence signals at 530 nm upon US irradiation, demonstrating the sufficient ^1^O_2_ generation (Figure S7, Supporting Information). 2,2,6,6‐tetramethylpiperidide (TEMP) is a spin trapper that was selected to serve as a ^1^O_2_ probe. A characteristic triple peak (1:1:1) of TEMP/^1^O_2_ adduct was observed directly when Ce6@Cu NPs were irradiated with US, as shown in the electron spin resonance (ESR) spectra (Figure 2b). The GSH depletion capability of Ce6@Cu NPs assessed using an indicator 5,5′‐dithiobis (2‐nitrobenzoic acid) (DTNB) revealed that Ce6@Cu NPs effectively depleted GSH, ascribed to satisfactory catalytic performance of Cu^2+^ (Figure 2c). Moreover, the GSH depletion capability was further strengthened when Ce6@Cu NPs was exposed to US irradiation, obtaining the desirable ROS generation (Figure S8, Supporting Information). These results confirmed that Ce6@Cu NPs possessed an excellent sonodynamic effect and GSH depletion capability.

In vivo cytotoxicity was evaluated via various assays, taking advantages of the on profoundenormous GSH depletion and SDT effects. The uptake of Ce6@Cu NPs by tumor cells investigated using confocal laser scanning microscopy (CLSM) revealed a time‐dependent increase in Ce6 fluorescence signals in U87MG cells, with the uptake rate of free Ce6 being lower than that of Ce6@Cu NPs. (Figure 2d,e; Figures S9 and S10, Supporting Information). The uptake of Ce6@Cu NPs by U87MG cells was further studied by ICP‐MS, which confirmed that U87MG cells effectively internalized Ce6@Cu NPs via endocytosis (Figure S11, Supporting Information). Additionally, the colocalization assay demonstrated that some Ce6@Cu NPs escaped from the lysosomes to the cytoplasm, leading to GSH depletion (Figure S12, Supporting Information). Then, the cytotoxicity of Ce6@Cu NPs on U87MG cells determined using a cell counting kit 8 (CCK‐8) showed a slight inhibition of U87MG cell growth by Ce6@Cu NPs due to the outstanding catalytic performances involving oxidative stress (Figure 2f). Furthermore, Ce6@Cu NPs exhibited significant inhibition of U87MG cell growth when exposed to US irradiation attributed to the enormous GSH depletion and the initiation of sonodynamic ROS storms. The propidium iodide (PI)/calcein‐AM co‐staining assay was selected to obtain a visual observation of the antitumor effect of Ce6@Cu NPs by CLSM and the images showed that Ce6@Cu NPs, when irradiated with US, exerted a higher cell‐killing effect compared to Ce6 plus US irradiation (Figure 2g; Figure S13, Supporting Information), in accordance to CCK8 results. The inhibitory effect of Ce6@Cu NPs on cell growth was further quantitatively evaluated by flow cytometry analysis. As anticipated, Ce6@Cu NPs combined with US irradiation triggered the highest percentage of U87MG cell death (Figure 2h). Collectively, these results provided a substantial support for catalytic‐enhanced SDT of Ce6@Cu NPs.

**Figure 3:**
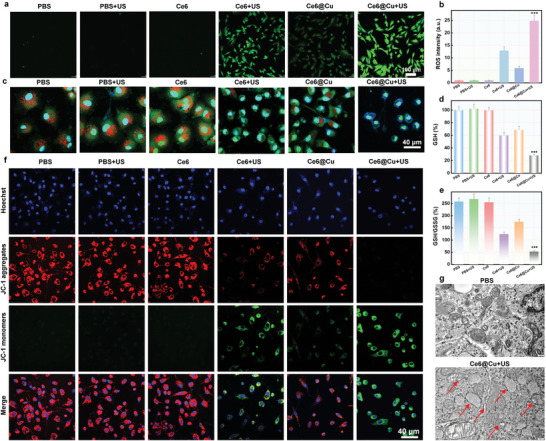
Cell death pathway induced by Ce6@Cu NPs. a) CLSM images of DCF‐stained tumor cells following treated with various formulations for 4 h and b) the corresponding quantification of fluorescence intensity. c) CLSM images of acridine orange (AO)‐stained U87MG cells after 24 h of incubation with various formulations. d) GSH level in tumor cells exposed to various formulation for 24 h. e) GSH/GSSG levels in U87MG cells exposed to various formulations. f) CLSM images of JC‐1‐stained cancer cells after the incubation with various formulations for 24 h. g) Bio‐TEM images of mitochondrial morphology following 24 h treatment with Ce6@Cu NPs plus US irradiation (red arrows indicate mitochondrial damage). *n* = 5, P‐values are calculated using the Student's two‐tailed *t*‐test, ^***^ *p* < 0.001.

The pathway regulating U87MG cell death was studied to discern the potential mechanism underlying the antitumor efficacy induced by Ce6@Cu NPs. First, the excellent GSH depletion capability and sonodynamic effect of Ce6@Cu NPs efficiently amplified cellular ROS level, which were evaluated using fluorescence probe DCFH‐DA.^[^ ^52^ ^]^ As expected, the combination of Ce6@Cu NPs with US irradiation resulted in a stronger green fluorescence intensity compared to that of Ce6 combined with US irradiation (**Figure** **3**a,b), consequently resulting in a synergistic catalytic therapy and sonodynamic cytotoxicity. Excessive ROS in tumor cells can induce disruption of lysosome membranes. CLSM images in Figure 3c and Figure S14 (Supporting Information) show that Ce6@Cu NPs combined with US irradiation, effectively reduced the red fluorescence signal, indicating a significant lysosomal damage and subsequent escape of Ce6@Cu NPs from the lysosome. The escaped Ce6@Cu NPs effectively depleted GSH and converted Cu^2+^ into Cu^+^. This dysfunction further was intensified by US irradiation (Figure 3d,e). As the mitochondrion is the primary target of Cu‐triggered cell death, the polarization in mitochondrial membrane potential (MMP) was examined using the fluorescence probe 5,5’,6,6’‐tetrachloro‐1,1’,3,3’‐tetraethyl‐imidacarbocyanine iodide (JC‐1), which monitored the shift in fluorescence from red to green. The CLSM images (Figure 3f) showed that the red fluorescence signal was reduced in the Ce6@Cu NPs group, while green fluorescence signal strengthened, demonstrating the damage of MMP. However, Ce6@Cu NPs caused an increase of the green fluorescence and a reduction in red fluorescence when combined with US irradiation, suggesting the significant MMP polarization. Moreover, biological transmission electron microscope (Bio‐TEM) was selected for a more in‐depth assessment of mitochondrial damage revealed a noticeable shrinkage of mitochondria and decreased cristae or even disappeared in U87MG cells treated with Ce6@Cu NPs and exposed to US irradiation, all characteristics of ferroptosis or cuproptosis (Figure 3g). These findings suggested that Ce6@Cu NPs induced cell death through a sonodynamic‐triggered combination of ferroptosis and cuproptosis.

**Figure 4:**
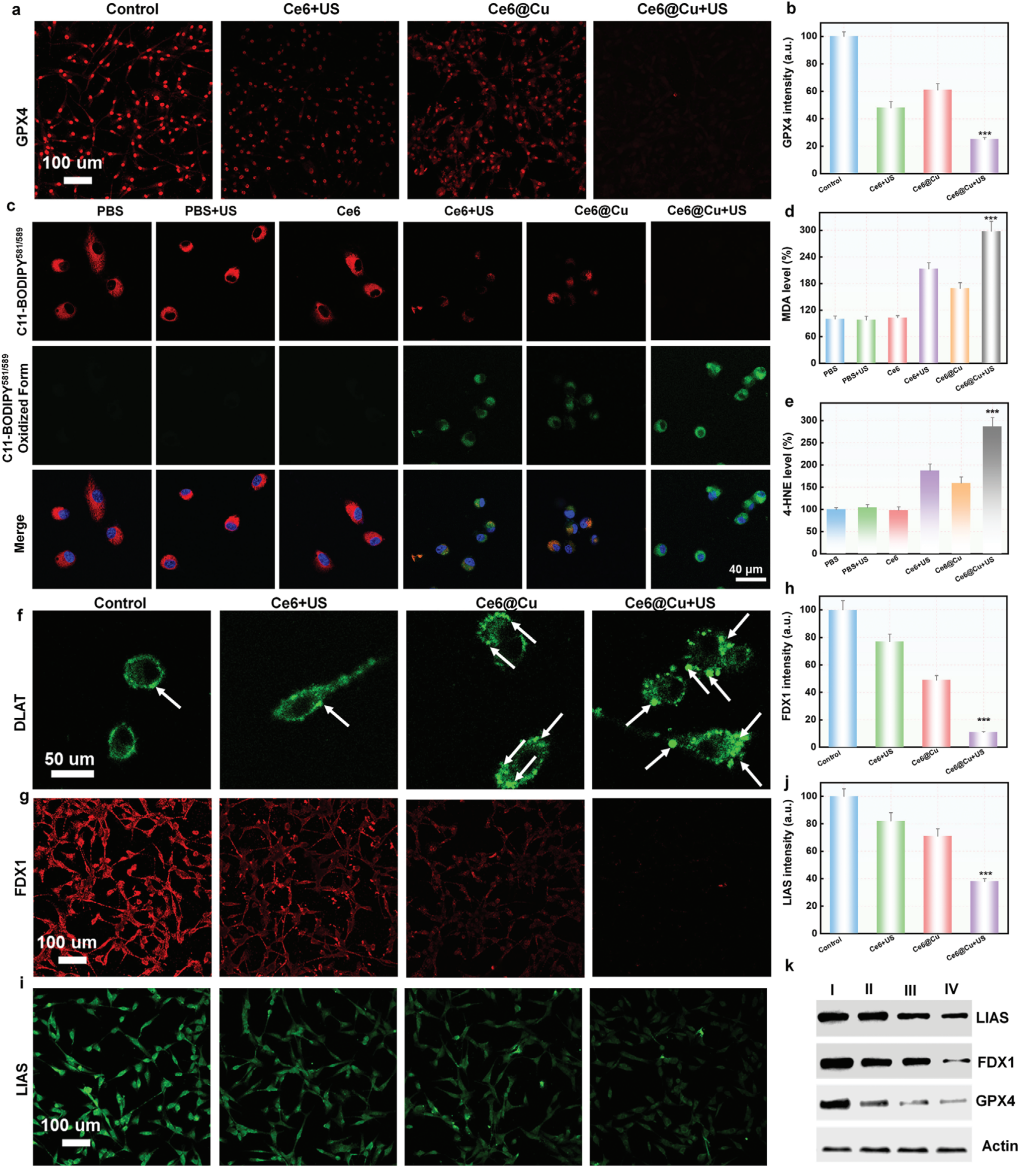
Ferroptosis and cuproptosis induced by Ce6@Cu NPs. a) CLSM images of GPX4 expression and b) the corresponding quantification in U87MG cells incubated with various formulations for 24 h. c) CLSM images of C11‐BODIPY^581/589^‐stained tumor cells cultured with different formulations for 24 h. d) MDA and e) 4‐HNE levels in U87MG cells treated with various formulations for 24 h. f) CLSM images of the oligomerization of lipoylated DLAT in U87MG cells treated with various formulations. g) CLSM images depicting FDX1 expression, and h) the associated quantification in U87MG cells after the treatment with various formulations for 24 h. i) CLSM images depicting LIAS expression, and j) the associated quantification in U87MG cells following a 24‐h incubation with various formulations. k) Western blot of GPX4, FDX1, and LIAS expressions in U87MG cells cultured with various formulations. I (Control), II (Ce6+US), III (Ce6@Cu), IV (Ce6@Cu+US). *n* = 5, P‐values are calculated using the Student's two‐tailed *t*‐test, ^***^ *p* < 0.001.

ROS oxidize susceptive PUFA, resulting in irreversible LPO. However, the LPO bioprocess in tumor cells is quickly relieved by endogenous GPX4 system. As previously reported, the depletion of GSH efficiently downregulate the GPX4 expression, resulting in ferroptosis. CLSM images revealed that Ce6@Cu NPs partially inhibited the GPX4 expression (**Figure** **4**a,b; Figure S15, Supporting Information), further strengthening the inhibitory effect when exposed to US irradiation. In addition, intrinsic characteristic of ferroptosis were studied to further confirm the ferroptotic mechanism triggered by Ce6@Cu NPs. The fluorescence indicator C11‐BODIPY^581/589^ was conducted to evaluate the Ce6@Cu NPs‐induced membrane LPO based on the fluorescence change from red to green. CLSM images (Figure 4c) showed significantly enhanced green fluorescence signals and reduced red fluorescence signals in the group treated with Ce6@Cu NPs (Figure 4c), suggesting the accumulation of lipid peroxides induced by Ce6@Cu NPs, greatly promoting ferroptosis. Moreover, the use of ferroptosis indicators such as malondialdehyde (MDA) and 4‐hydroxynonenal (4‐HNE) chosen to confirm Ce6@Cu NPs‐induced ferroptosis resulted in a significant increase in their levels in U87MG cells treated with Ce6@Cu NPs and exposed to US irradiation (Figure 4d,e). Consequently, these findings provided evidences that Ce6@Cu NPs efficiently induced ROS generation, GSH depletion, and the inhibition of GPX4 expression, leading to irreversible ferroptosis.

Previous studies reported a strong association between cuproptosis and mitochondrial damage.^[^ ^4^ ^]^ Thus, the accumulation of Ce6@Cu NPs caused a high Cu concentration in U87MG cell, resulting in cuproptosis. Cu^+^ binds to lipoylated mitochondrial enzymes, causing an aggregation of lipoylated DLAT. CLSM images demonstrated that Ce6@Cu NPs effectively induced the aggregation of DLAT when exposed to US irradiation (Figure 4f; Figure S16, Supporting Information), thereby initiating the cuproptosis pathway. Moreover, the oligomerization of DLAT feedback downregulates the upstream FDX1 protein.^[^ ^18^ ^]^ Immunofluorescence (IF) results showed that the expression of FDX1 in tumor cells treated with Ce6@Cu NPs plus US irradiation was completely inhibited (Figure 4g,h; Figure S17, Supporting Information). FDX1 is involved in the TCA cycle, and its downregulation resulted in the inhibition of downstream proteins such as LIAS, as proved by CLSM images (Figure 4i,j; Figure S18, Supporting Information). Western blot results further validated the significant inhibition of GPX4, FDX1, and LIAS expressions induced by Ce6@Cu NPs combined with US irradiation. (Figure 4k). Taken together, these results confirmed that Ce6@Cu NPs effectively induced tumor cuproptosis via the aggregation of DLAT and the downregulation of LIAS and FDX1.

**Figure 5:**
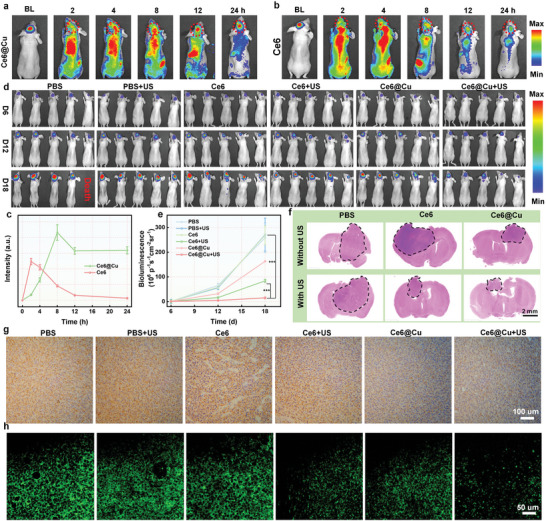
In vivo antitumor effect of Ce6@Cu NPs. a) In vivo fluorescence images of orthotopic U87MG‐Luc tumor‐bearing mice after the intravenous administration of Ce6@Cu NPs, b) free Ce6, and c) the corresponding quantification of fluorescence signals. d) BL images and e) the corresponding BL signals in mice injected with different formulations at day 12, and 18 post‐U87MG tumor implantation. f) H&E staining of brain collected from different groups. g) Immunochemical staining of FDX1 expression in different group. h) Immunofluorescent staining to assess the expression of GPX4. *n* = 5, P‐values are calculated using the Student's two‐tailed *t*‐test, ^***^ *p* < 0.001.

The Ethical Committee of Fujian Medical University approved the protocol (IACUC FJMU2022‐0608) to perform animal experiments. Biochemical factors were selected to assess the biocompatibility of Ce6@Cu NPs. No significant changes in blood indices were observed after the intravenous injection of Ce6@Cu NPs (Figure S19, Supporting Information). Hematoxylin and eosin (H&E) staining did not show any noticeable damage in the major organs (Figure S20, Supporting Information), offering a substantial evidence of the outstanding biosafety of Ce6@Cu NPs. Subsequently, an orthotopic U87MG‐Luc glioblastoma mouse model was established, as evidenced by bioluminescence (BL) results. Fluorescence images unveiled that Ce6@Cu NPs demonstrated a superior ability to penetrate the BBB compared to free Ce6 (**Figure** **5**a–c). This resulted in an impressive accumulation efficacy in the tumor sites due to the enhanced permeability and retention (EPR) effect. The biodistribution of Ce6@Cu NPs in tumor‐bearing mice further confirmed the satisfactory tumor accumulation efficiency (Figures S21 and S22, Supporting Information), significantly implying the successful therapeutic effect of sonodynamic‐amplified ferroptosis and cuproptosis.

The combined therapeutic effectiveness of ferroptosis and cuproptosis induced by Ce6@Cu NPs was evaluated in orthotopic U87MG‐Luc tumor‐bearing mice. The orthotopic U87MG‐Luc mice were randomly assigned into six groups: PBS, PBS plus US, Ce6, Ce6 plus US, Ce6@Cu NPs, and Ce6@Cu NPs plus US. Figure 5d,e illustrates that Ce6 partially inhibited tumor growth after US irradiation, attributed to the great sonodynamic effect. Ce6@Cu NPs also showed a modest suppression of tumor growth, ascribed to the synergistic ferroptosis and cuproptosis involving GSH consumption. Moreover, the antitumor efficacy was significantly intensified after US irradiation due to sonodynamic‐amplified ROS generation, considerably improving the survival rate of tumor‐bearing mice (Figure S23, Supporting Information). The mice in PBS group gradually lost weight due to the interference of glioblastoma growth with daily life (Figure S24, Supporting Information), while no noticeable change in body weight was observed in the Ce6@Cu NPs plus US group, attributing the outstanding therapeutic effect. The successful therapeutic efficacy was confirmed through H&E, terminal deoxynucleotidyl transferase‐mediated dUTP‐biotin nick end labeling (TUNEL), Ki67, immunohistochemistry, and IF staining of the excised brain. H&E images (Figure 5f; Figure S25, Supporting Information) showed that the mice in the Ce6@Cu NPs group had the smallest tumor volume when exposed to US irradiation. TUNEL staining results showed that mice treated with Ce6@Cu NPs plus US suffered the most severe tumor cell death (Figure S26, Supporting Information), confirming the profound therapeutic efficacy. Ki67 results illustrated the presence of the lowest tumor proliferation rate in mice treated with Ce6@Cu NPs plus US irradiation (Figure S27, Supporting Information), consistent with TUNEL results. Immunohistochemistry and IF staining results demonstrated that Ce6@Cu NPs induced the lowest expressions of GPX4, FDX1, and LIAS (Figure 5g,h; Figures S28 and S29, Supporting Information), indicating the induction of ferroptosis and cuproptosis. Furthermore, the concentration of copper ions in the tumor was measured using ICP‐MS. The experimental results indicated that intravenous injection of Ce6@Cu NPs effectively elevated the copper ion content within glioblastoma lesion, facilitating the induction of cuproptosis in the tumors (Figure S30, Supporting Information). These findings verified the synergistic ferroptosis and cuproptosis induced by carrier‐free Ce6@Cu NPs and emphasized the potential for clinical translation.

## 3 Conclusion

Carrier‐free nanoparticles (Ce6@Cu NPs) self‐assembled through the coordination of Cu^2+^ ions with Ce6, promoted sonodynamic effect for the synergy of ferroptosis and cuproptosis. Ce6@Cu NPs internalized into U87MG cells exerted a remarkable sonodynamic effect in generating ^1^O_2_ under US irradiation. Ce6@Cu NPs also depleted the high level of endogenous GSH, resulting in GPX4 inactivation, promoting LPO, and increasing irreversible ferroptosis. Furthermore, the Cu^+^ concentration in tumor cells, generated by the reaction of Cu^2+^ with GSH, significantly increased, thus inhibiting the expressions of FDX1 and LIAS, triggering DLAT aggregation, and inducing cuproptosis in return. Additionally, Ce6@Cu NPs demonstrated an impressive capacity to penetrate the BBB, causing the remarkable accumulation efficacy in the tumor. Ce6@Cu NPs effectively suppressed tumor growth in vivo with negligible side effects thanks to the synergistic effect of ferroptosis and cuproptosis. This work provided a proof‐of‐concept for a valid approach triggering the combination of ferroptosis and cuproptosis through catalytic‐enhanced SDT.

## Conflict of Interest

The authors declare no conflict of interest.

## Supporting information

Supporting Information

## Data Availability

The data that support the findings of this study are available in the supplementary material of this article.
