# Correction to “Carrier‐Free Self‐Assembly Nano‐Sonosensitizers for Sonodynamic‐Amplified Cuproptosis‐Ferroptosis in Glioblastoma Therapy”

**DOI:** 10.1002/advs.202521439

**Published:** 2025-11-08

Yang Zhu,* Xuegang Niu, Chengyu Ding, Yuanxiang Lin, Wenhua Fang, Lingjun Yan, Junjie Cheng, Jianhua Zou, Yu Tian, Wei Huang, Wen Huang, Yuanbo Pan, Tiantian Wu,* Xiaoyuan Chen,* and Dezhi Kang*. Carrier‐Free Self‐Assembly Nano‐Sonosensitizers for Sonodynamic‐Amplified Cuproptosis‐Ferroptosis in Glioblastoma Therapy. *Adv Sci*, 2024, 23, 2402516.

A wrong image was included in the Ce6@Cu+US group of lung H&E staining in Figure S20. We have replaced the misused image in Figure S20 with the correct one:


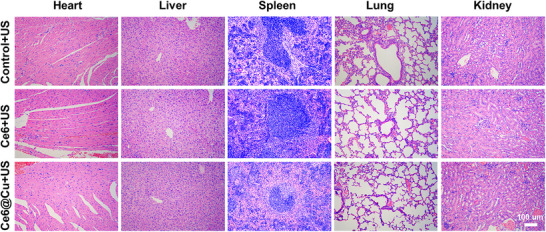


**Figure S20**. Haematoxylin and eosin (H&E)‐stained images of major organs harvested from different groups of mice at 12 days post‐treatment.]

These errors do not affect the results or conclusions of the paper.

We apologize for any inconvenience caused by these errors.

