# Supplementary material for: Carrier‐Free Self‐Assembly Nano‐Sonosensitizers for Sonodynamic‐Amplified Cuproptosis‐Ferroptosis in Glioblastoma Therapy
Source: Adv Sci (Weinh). 2024 Apr 6;11(23):2402516. doi: 10.1002/advs.202402516 (PMC11187904; doi:10.1002/advs.202402516)
Supplement: Supplementary file 1 — Supporting Information [file ADVS-11-2402516-s001.pdf]

ADVANCED SCIENCE
Open Access

Supporting Information

for *Adv. Sci.*, DOI 10.1002/advs.202402516

Carrier-Free Self-Assembly Nano-Sonosensitizers for Sonodynamic-Amplified Cuproptosis-Ferroptosis in Glioblastoma Therapy

Yang Zhu, Xuegang Niu, Chengyu Ding, Yuanxiang Lin, Wenhua Fang, Lingjun Yan, Junjie Cheng, Jianhua Zou, Yu Tian, Wei Huang, Wen Huang, Yuanbo Pan, Tiantian Wu*, Xiaoyuan Chen* and Dezhi Kang**

Supporting Information

Carrier-Free Self-Assembly Nano-Sonosensitizers for Sonodynamic-Amplified Cuproptosis-Ferroptosis in Glioblastoma Therapy

Yang Zhu, Xuegang Niu, Chengyu Ding, Yuanxiang Lin, Wenhua Fang, Lingjun Yan, Junjie Cheng, Jianhua Zou, Yu Tian, Wei Huang, Wen Huang, Yuanbo Pan, Tiantian Wu*, Xiaoyuan Chen*, Dezhi Kang**

Experimental Procedures

Chemicals

Copper chloride ($CuCl_2$), chlorin e6 (Ce6), paraformaldehyde (PFA), Tween 20, Triton X-100 were purchased from Sinopharm Chemical Reagents (Shanghai, China). 5,5'-dithiobis (2-nitrobenzoic acid) (DTNB), and C11-BODIPY$^{581/591}$ were provided by Sigma-Aldrich (St. Louis, USA). Hoechst, Calcein-AM, propidium iodide (PI), 2',7'-dichlorofluorescin diacetate (DCFH-DA), cell count kit-8 (CCK-8), annexin V-FITC/PI apoptosis detection kit, acridine orange (AO), and 1,1',3,3'-tetraethyl-5,5',6,6'-tetrachloroimidacarbocyanine iodide (JC-1) were bought from Beyotime (Shanghai, China). Dulbecco's modified eagle medium (DMEM) was purchased from Hyclone (Logan, USA). 2,2,6,6-tetramethylpiperidide (TEMP) was bought from Dojindo (Dojindo, China). All antibodies (glutathione peroxides 4 (GPX4), dihydrolipoamide S-acetyltransferase (DLAT), ferredoxin-1 (FDX1), and lipoyl synthase (LIAS)) were bought from Abcam. Deionized (DI) water was obtained from a Milli-Q water purification system.

Instruments

Powder X-ray diffraction patterns of samples were recorded on a Rigaku Miniflex-600. Transmission electron microscopy (TEM) images were taken by Hitachi-7700. X-ray photoelectron spectroscopy (XPS) was collected on scanning X-ray microprobe (PHI 5000 Verasa, ULAC-PHI). Fluorescence imaging by using confocal laser scanning microscopy (CLSM, Zeiss 800). The UV absorbance of samples were measured by ultraviolet visible (UV-Vis) spectrophotometer (Agilent). Metal content analysis by using inductively coupled plasma mass spectrometer (ICP-MS, PlasmaQuad 3,

Thermo Elemental). Dynamic light scattering (DLS) and Zeta potential were obtained by using a Brook haven. The cell death was analyzed by flow cytometer (CytoFLEX, Beckman).

Synthesis of Ce6@Cu nanoparticles (Ce6@Cu NPs)

The Ce6@Cu NPs were fabricated *via* a self-assembled strategy. 10 mg Ce6 was dissolved in dimethylsulfoxide (DMSO), and then 4 mL 10 mg/mL $CuCl_2$ were dropwise added into the Ce6 solution under mild stirring. After stirring for 24 h, the as-prepared Ce6@Cu NPs were dialyzed for 24 h.

ROS generated by Ce6@Cu NPs

The sonodynamic effect of Ce6@Cu NPs was measured using DCFH-DA as a probe. Briefly, Ce6@Cu NPs (20 μg/mL) and DCFH-DA (20 μM) were successively mixed, and the mixture was irradiated using ultrasound (US) (1 W/cm^2) for 5 min. Then the fluorescence spectra were measured.

To further confirm the Ce6@Cu NPs-catalyzed $^1O_2$ generation, electron spin resonance (ESR) analysis was employed using TEMP as the spin trapper. 40 μg/mL Ce6@Cu NPs, and 10 mM TEMP was irradiated using US (1 W/cm^2) for 5 min. Then, the mixture was transferred to a quartz tube for ESR measurement.

GSH depletion capability of Ce6@Cu NPs

The GSH depletion capacity of Ce6@Cu NPs was evaluated using a probe DTNB. 1 mM GSH, 100 μg/mL Ce6@Cu NPs, and 1 mM DTNB incubated for 60 min. Then the absorption spectra were analyzed.

Cellular uptake

U87MG cells were seeded in confocal dishes for 12 h. After 4 h of incubation with Ce6@Cu NPs, tumor cells were co-stained with 10 μM Hoechst and 10 μM Lyso-tracker for 20 min. The fluorescence imaging of U87MG cells was imaged by CLSM.

Cytotoxicity assessments

Cell-viability was measured by the CCK-8 assay, live/dead cell staining assay, and flow cytometry analysis. For CCK-8 assay, U87MG cells were planted for 24 h. Then, the cells were incubated with various concentrations of Ce6 and Ce6@Cu NPs without/with US irradiation. After treatment for 24 h, the medium was replaced with fresh medium containing 10 μL CCK-8 and quantified by the absorbance at 450 nm using a microplate reader.

Live/dead cell staining assay was monitored by CLSM to observe the toxicity. U87MG cells were seeded in confocal dishes and incubated for 12 h. After 24 h of exposure to Ce6@Cu NPs without/with US irradiation (1 W/cm^2), the cells were co-stained with calcein-AM and PI for 20 minutes. The fluorescence imaging of cells was observed by confocal microscopy.

For analysis of cell death, Annexin V-FITC and PI kit was carried out. U87MG cells were seeded and incubated 12 h. Subsequently, the cells were exposed to Ce6@Cu NPs without/with US irradiation. After co-staining with Annexin V-FITC and PI according to the manufacturer's protocols. The quantitative cell death was determined by flow cytometry.

***In vitro* reactive oxygen species (ROS) generation**

U87MG cells were seeded in confocal dish. After incubation for 12 h, the cells were treated with different formulations for 4 h. Then, the cells were co-stained with DCFH-DA (10 μM) and Hoechst (10 μM). After 20 minutes of incubation, the medium was removed and the cells were washed with DMEM. The fluorescence imaging of cells was imaged by confocal microscopy.

Intracellular GSH and GSSG content

U87MG cells were plated in 6-well plates and incubated for 24 h. Subsequently, the cells were exposed to Ce6@Cu NPs without/with US irradiation. The GSH and GSSG contents were measured using a DTNB kit. The assay was carried out according to the manufacturer's instructions. The absorbance of 340 nm was measured by a microplate reader.

Change of mitochondrial membrane potential (MMP)

To investigate the MMP, U87MG cells were seeded and incubated for 24 h. Subsequently, the cells were exposed to Ce6@Cu NPs without/with US irradiation. Then the cells were treated according to the JC-1 kit. The fluorescence imaging of cells was analyzed by confocal microscopy.

Lipid peroxidation (LPO) initiated by Ce6@Cu NPs

The cellular LPO assay was carried out by using a BODIPY$^{581/591}$-C11 probe. U87MG cells were seeded and incubated for 24 h. Subsequently, the cells were exposed to Ce6@Cu NPs without/with US irradiation. Then the cells were stained with BODIPY$^{581/591}$-C11 probe and Hoechst for 30 min. The fluorescence imaging of cells was imaged by CLSM.

Intracellular GPX4, FDX1, LIAS expressions

U87MG cells were treated with Ce6@Cu NPs plus US irradiation. Subsequently, the cells were fixed with 1% PFA at 37 ºC for 15 min and then permeabilized with PBS buffer containing 0.2% Triton X-100 at 37 ºC for 10 min. After blocking with PBS buffer containing 0.05% Tween 20 and 10% goat serum at room temperature for 45 min, the cells were incubated with different antibodies (Alexa Fluor® 640/488 Conjugate) in a humidified chamber for 1 h. Finally, the fluorescence images were acquired to analyze the expression levels of GPX4, FDX1, LIAS. Moreover, the expression of GPX4, FDX1, LIAS was also measured by western blot assay.

Intracellular GPX4 activity assay

Intracellular GPX4 activity was measured using a commercial cellular glutathione peroxidase kit. Briefly, U87MG cells were planted in 6-well plates and incubated for 12 h. Then the cells were exposed to Ce6@Cu NPs without/with US irradiation. Cell processing and analysis followed the manufacturer's instructions.

Enzyme-linked immunosorbent assay

Commercially available enzyme-linked immunosorbent assay (ELISA) kits were used to measure the levels of 4-hydroxynonenal (4-HNE) and malondialdehyde (MDA) in the indicated samples according to the manufacturers' instructions.

***In vivo* antitumor efficacy**

Animal experiments were performed according to the protocol (IACUC FJMU2022-0608) approved by The Ethical Committee of Fujian Medical University. Orthotopic U87MG-Luc tumor-bearing mice were randomly divided into six groups (5 mice per group) and intravenously administrated with 2 mg/kg Ce6@Cu NPs. After 8 h post-injection, the three x exposure groups were irradiated with US (1 W/cm^2) for 10 min. After 12 days of treatment, the mice were euthanatized for histological examination.

Statistical analysis

All quantitative data were expressed as the mean ± standard deviation (SD). Statistical analyses were performed using the Student's two-tailed t-test (*P < 0.05, **P < 0.01, ***P < 0.001).

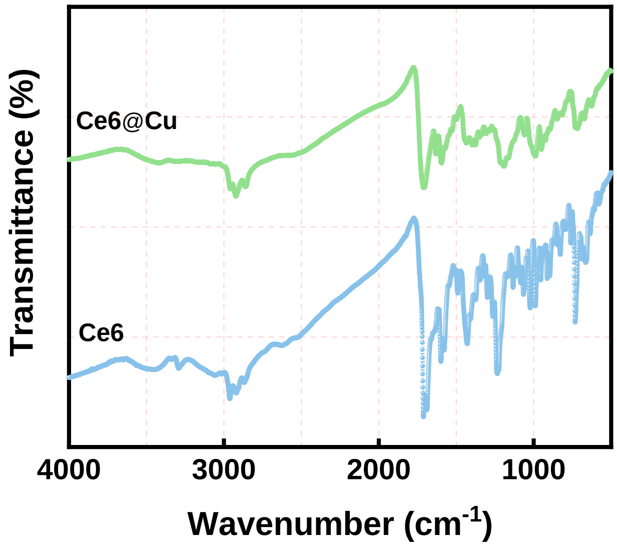

Figure S1. The Fourier transform infrared spectra of Ce6 and Ce6@Cu NPs.

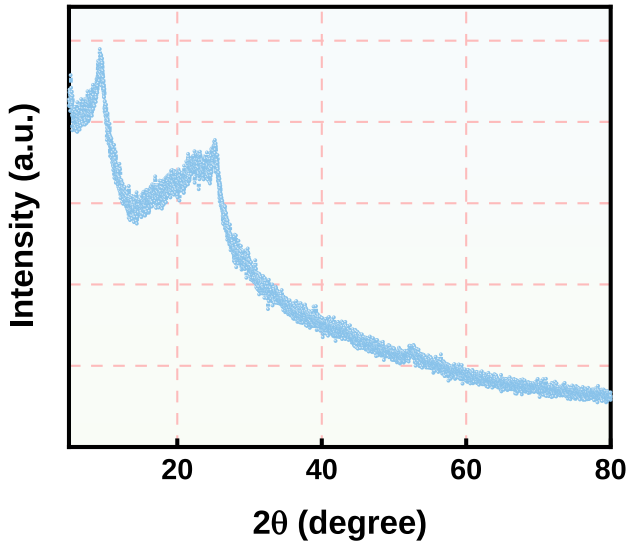

Figure S2. X-ray diffraction (XRD) spectrum of Ce6@Cu NPs.

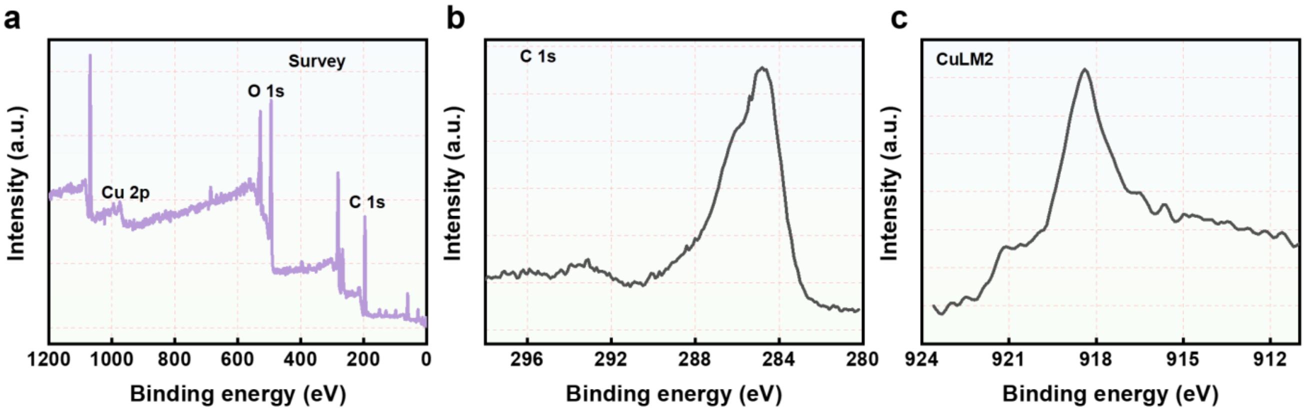

Figure S3. XPS spectra of Ce6@Cu NPs. a) Survey, b) C 1s, and c) CuLM2.

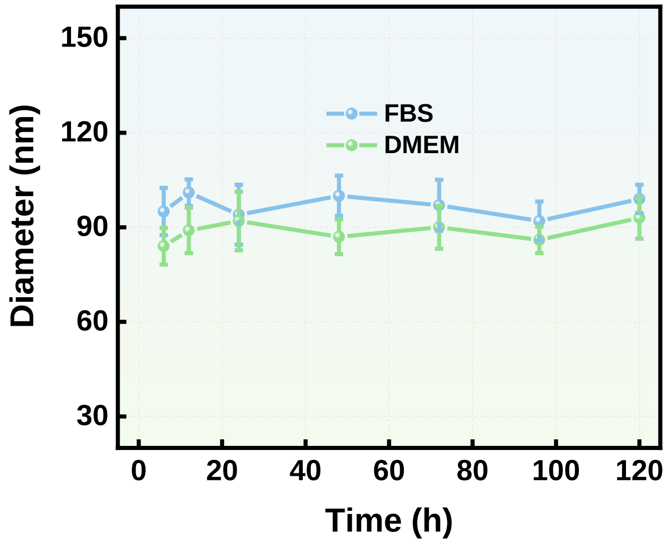

Figure S4. The stability of the Ce6@Cu NPs in DMEM and fetal bovine serum (FBS).

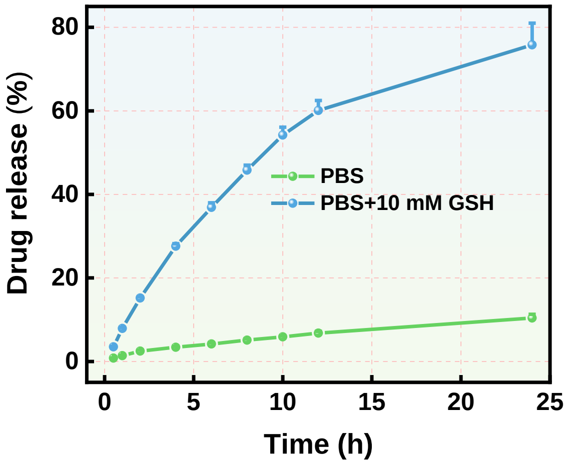

Figure S5. Drug release curve of Ce6@Cu NPs in different media.

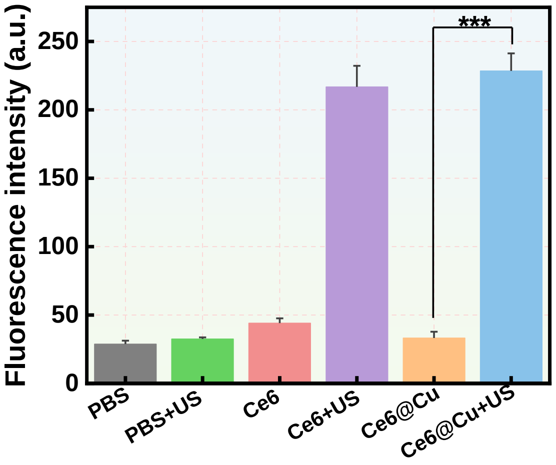

Figure S6. The corresponding quantification fluorescence intensity of DCFH-DA treated with Ce6@Cu NPs or Ce6 upon US irradiation.

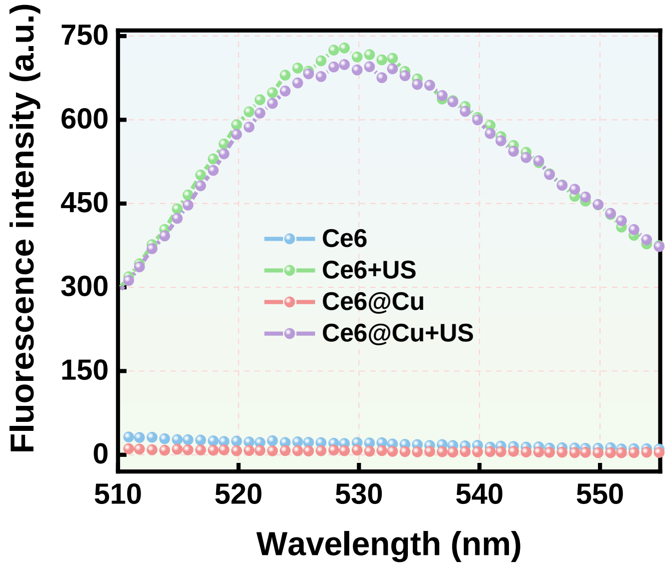

Figure S7. Fluorescence spectra of singlet oxygen sensor green (SOSG) treated with Ce6@Cu NPs or Ce6 upon US irradiation.

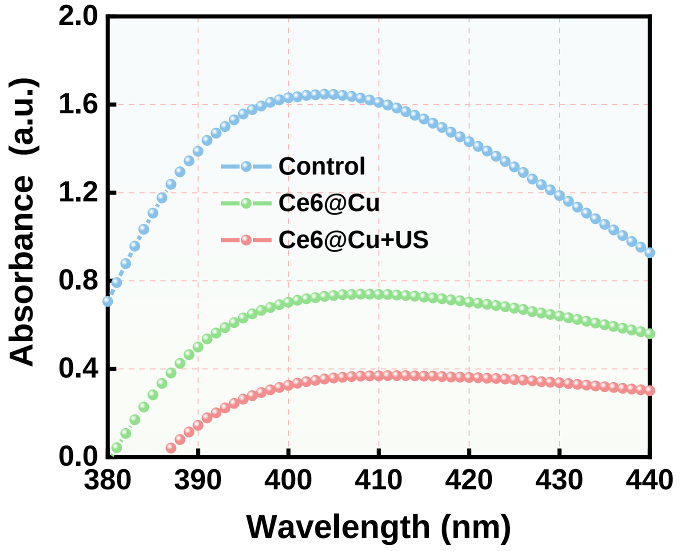

Figure S8. UV-Vis spectra of DTNB treated with Ce6@Cu NPs upon US irradiation.

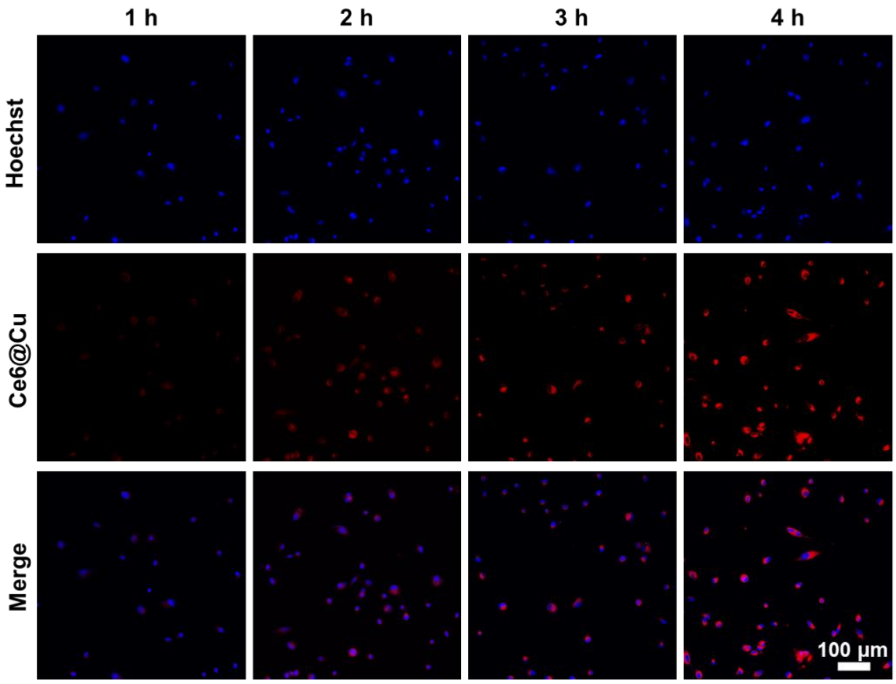

Figure S9. Fluorescence images of U87MG cells incubated with Ce6@Cu NPs at different time points.

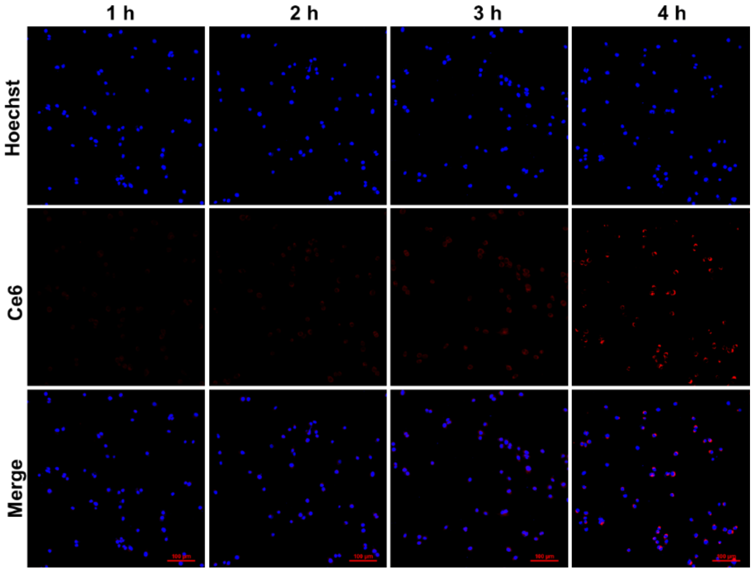

Figure S10. Fluorescence images of U87MG cells incubated with Ce6 at different time points.

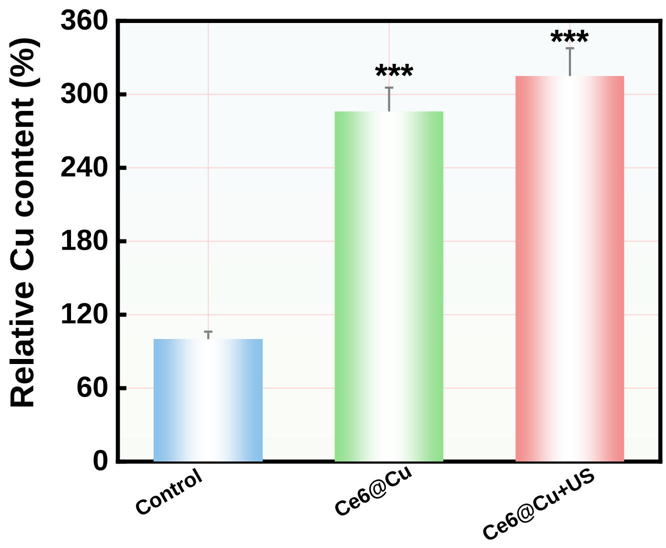

Figure S11. Bar graph showing the Cu content in U87MG cells incubated with Ce6@Cu NPs.

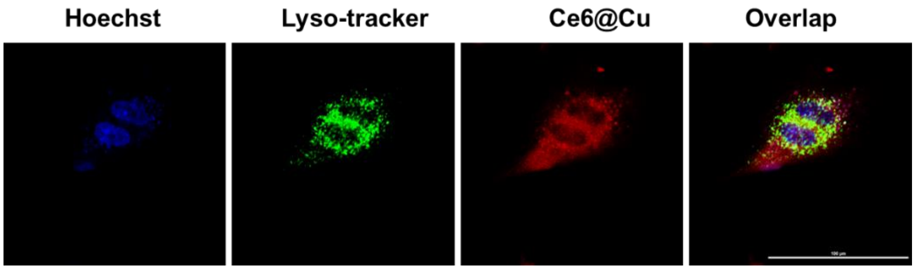

Figure S12. CLSM images of colocalization in U87MG cells between the lysosome tracker (green channel) and Ce6@Cu NPs (red channel) after 4 h of incubation.

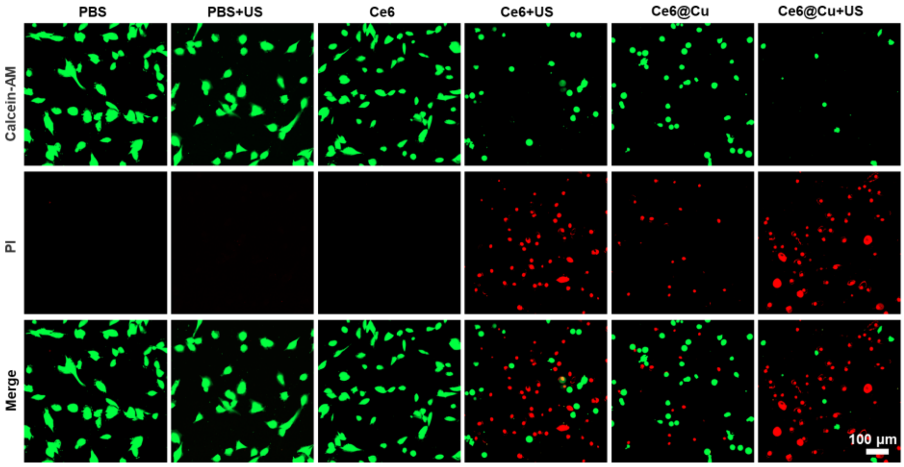

Figure S13. CLSM images demonstrated calcein-AM/PI co-staining of U87MG cells following 24 h incubation with various formulations.

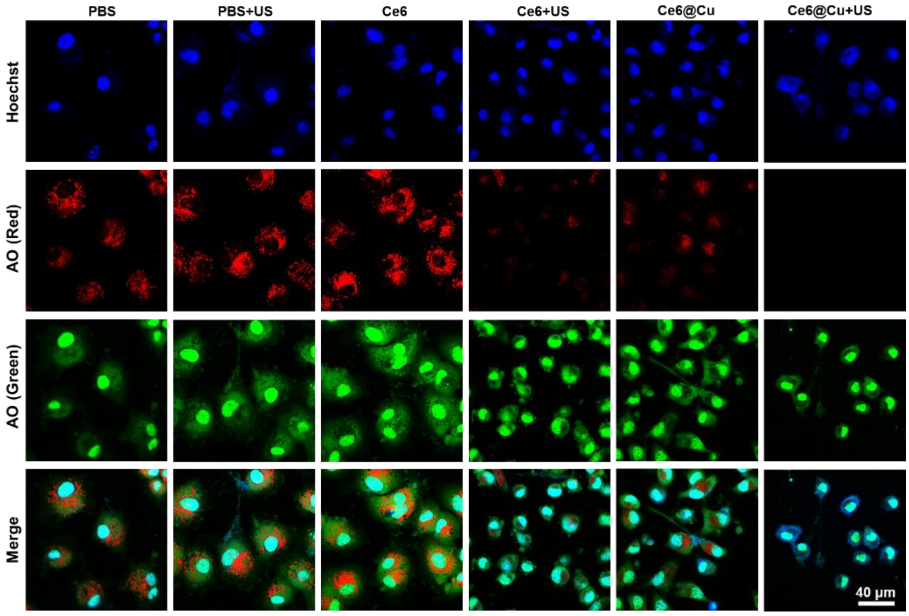

Figure S14. CLSM images of AO-stained U87MG cells after 24 h of incubation with various formulations.

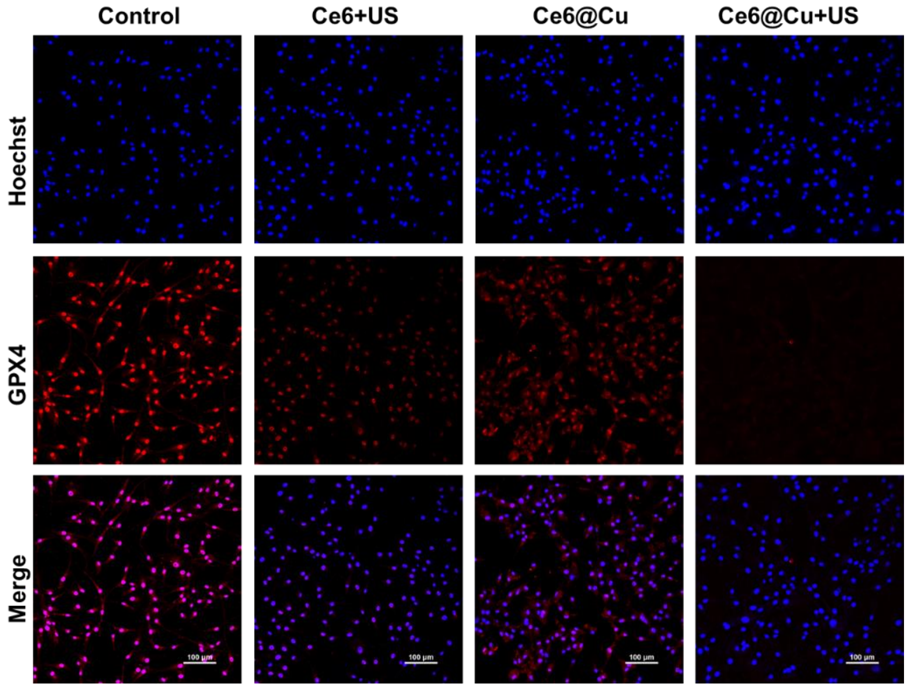

Figure S15. CLSM images of GPX4 expression in U87MG cells incubated with various formulations for 24 h.

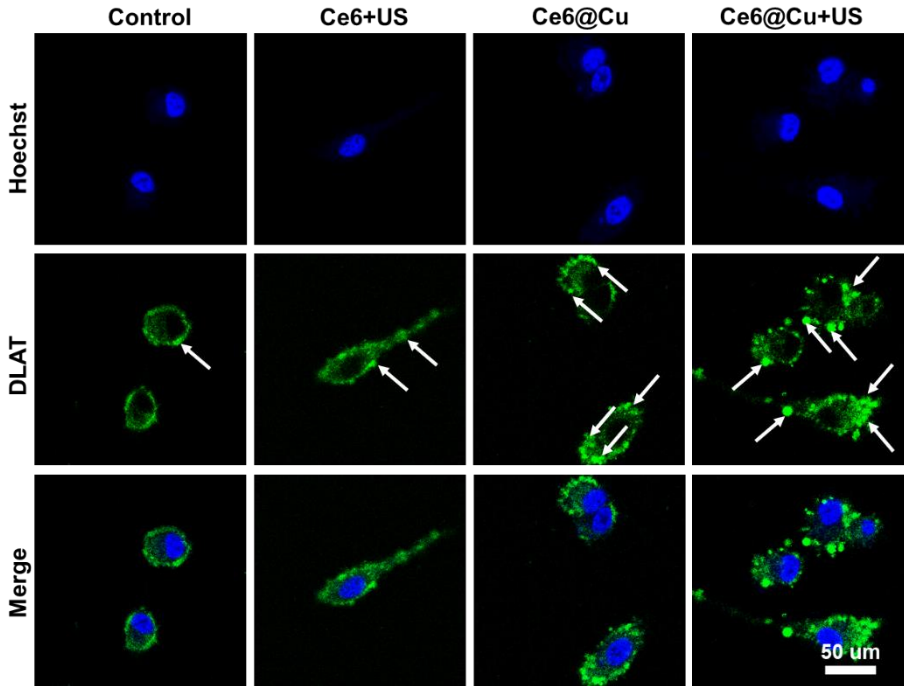

Figure S16. CLSM images of the oligomerization of lipoylated DLAT cultured with various formulations for 24 h.

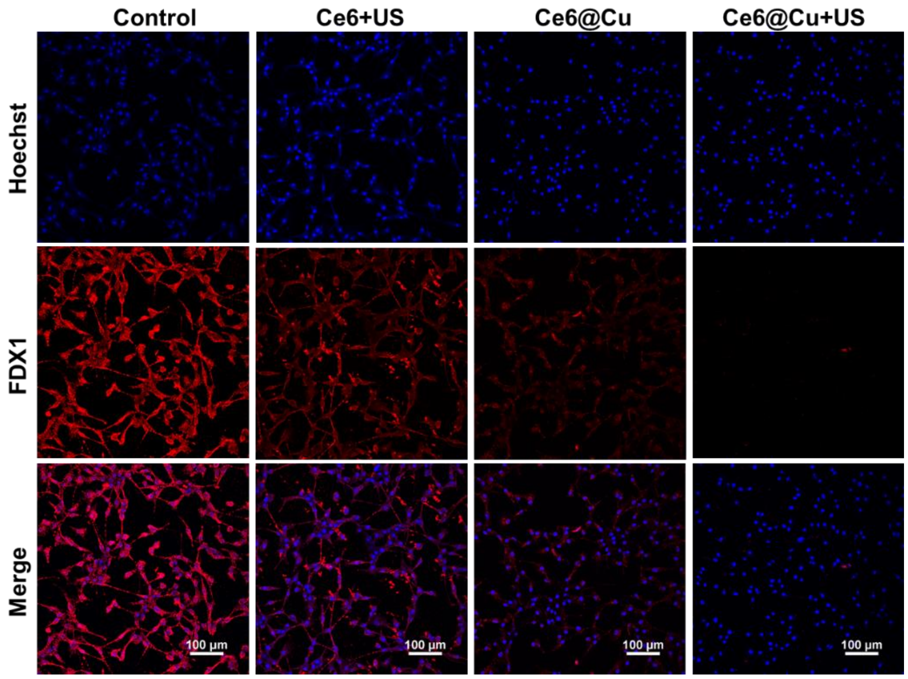

Figure S17. CLSM images depicting FDX1 expression in U87MG cells following a 24-hour incubation with various formulations.

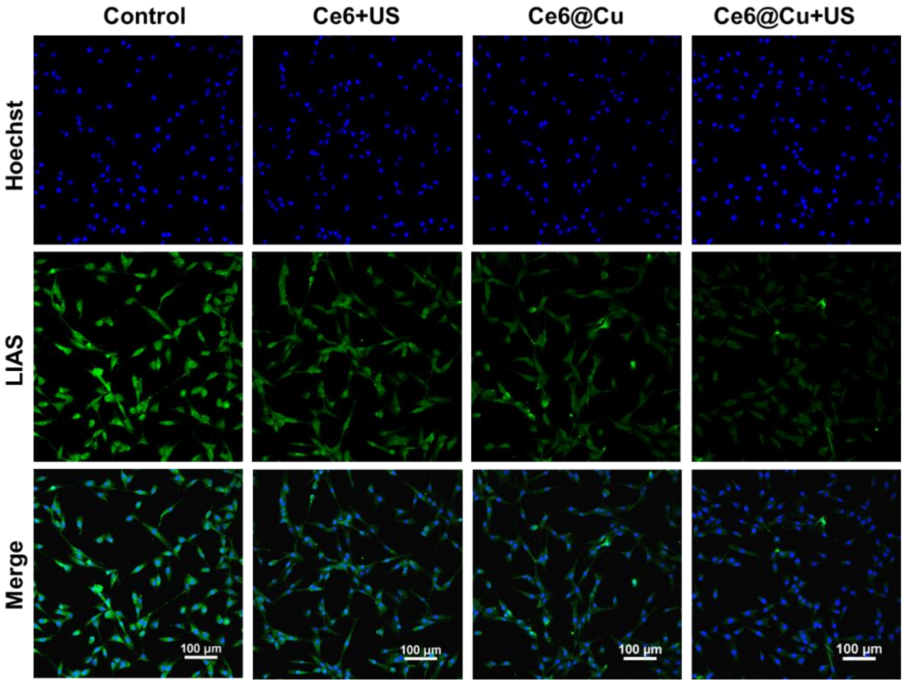

Figure S18. CLSM images depicting LIAS expression in U87MG cells following a 24-hour incubation with various formulations.

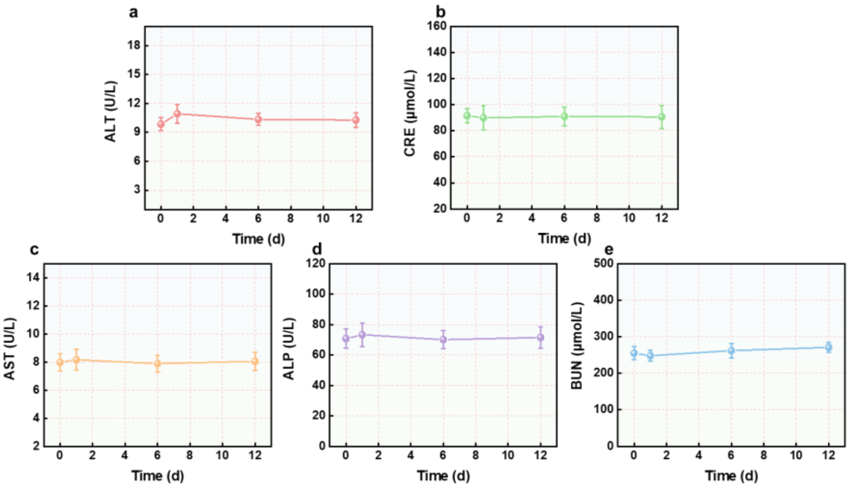

Figure S19. Blood biochemistry analysis of mice after intravenous injection with Ce6@Cu NPs at different time points. a) Alanine transaminase (ALT). b) Creatinine (CRE). c) Aspartate aminotransferase (AST). d) Alkaline phosphatase (ALP). (e) Blood urea nitrogen (BUN) levels at day 1, 6, and 12.

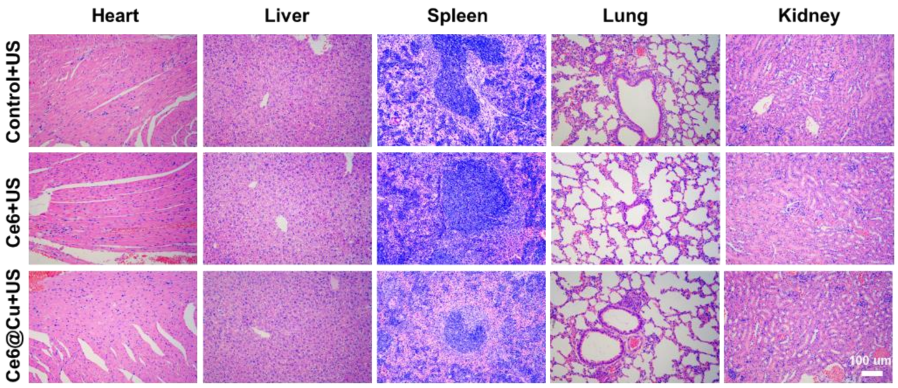

Figure S20. Haematoxylin and eosin (H&E)-stained images of major organs harvested from different groups of mice at 12 days post-treatment.

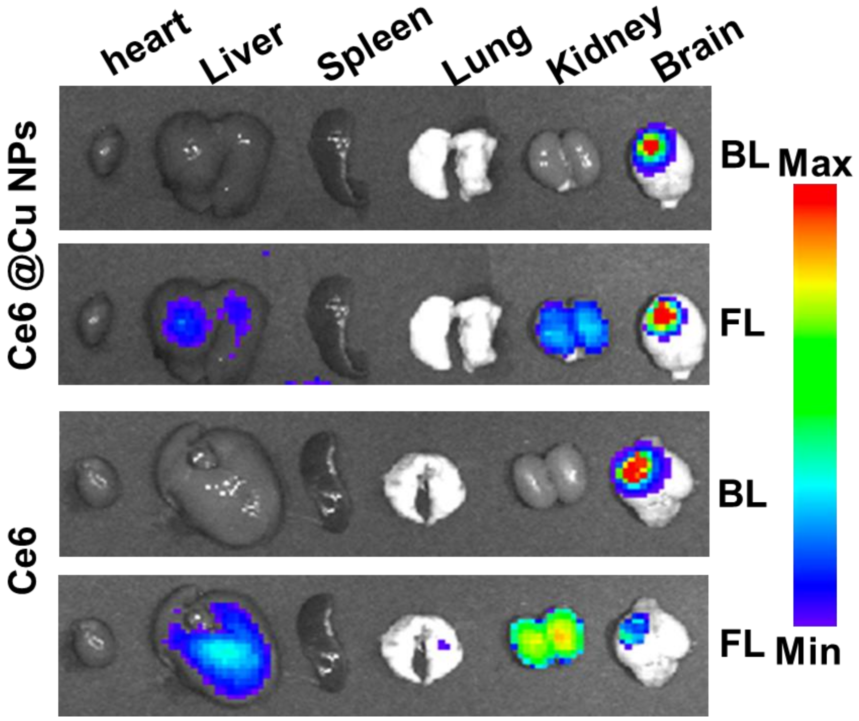

Figure S21. *EX vivo* fluorescence (FL) /bioluminescence (BL) images of major organs (heart, liver, spleen, lung, kidney, and brain) obtained from tumor-bearing mice 24 hours after injection with Ce6 or Ce6@Cu NPs.

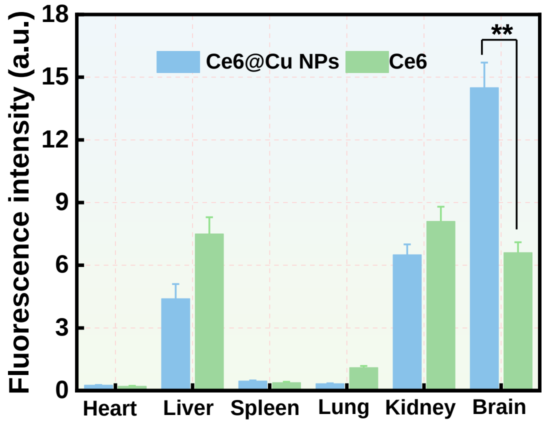

Figure S22. The corresponding quantification fluorescence intensity of the major organs (heart, liver, spleen, lung, kidney, and brain).

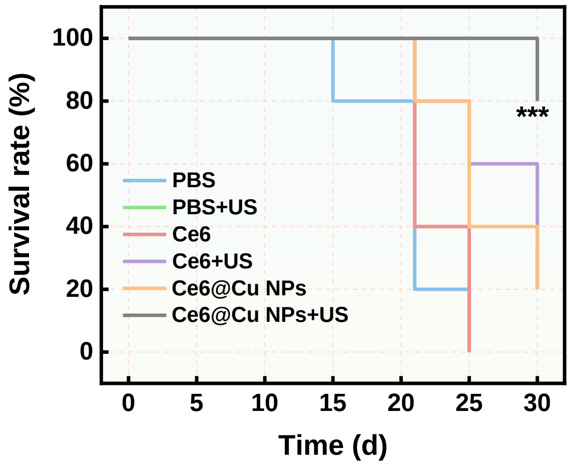

Figure S23. Kaplan-Meier survival curves for various groups of mice.

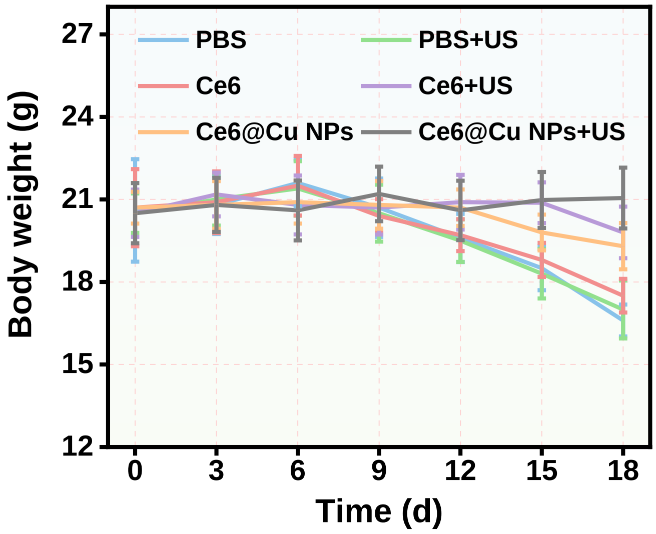

Figure S24. The body weights of mice bearing tumors during the treatment period.

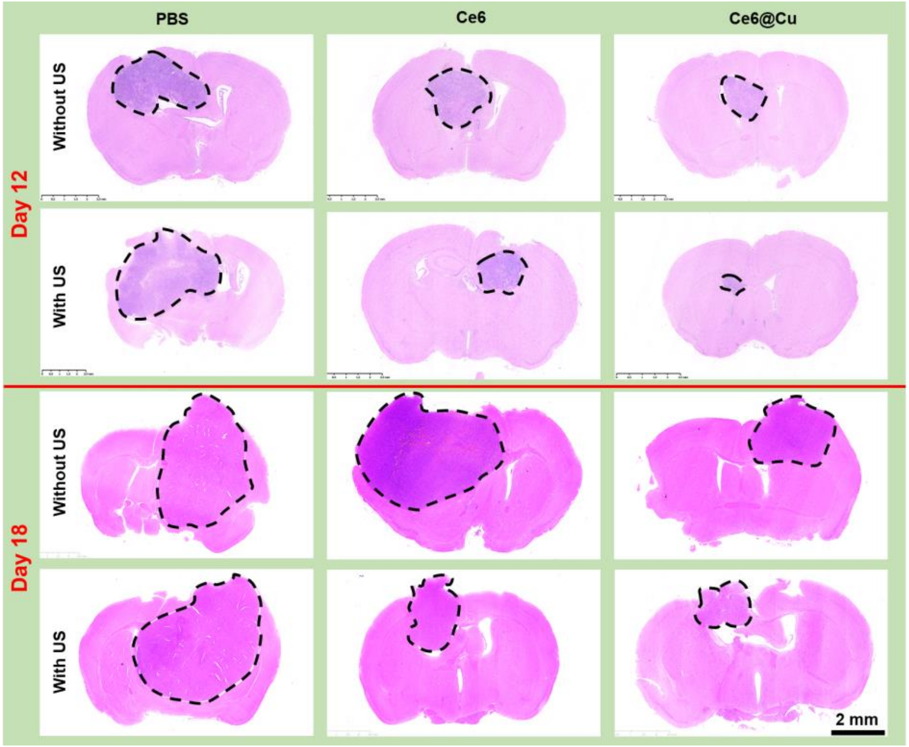

Figure S25. H&E staining was performed on brains collected from various groups at different time points.

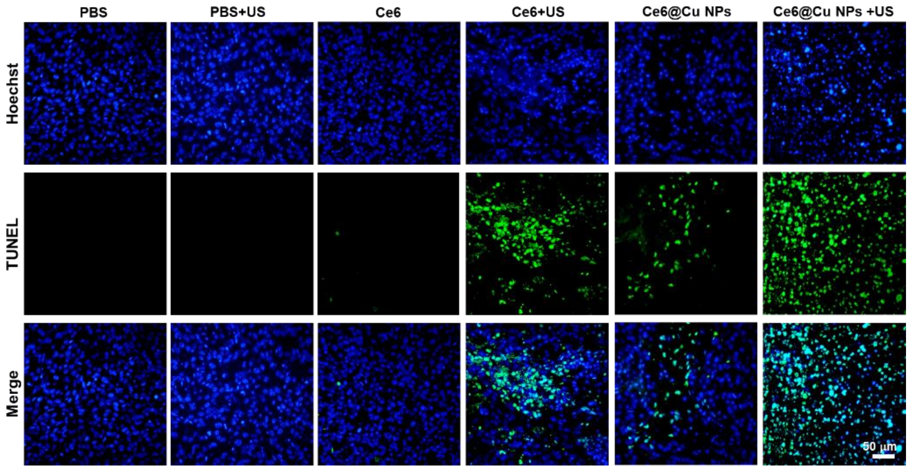

Figure S26. Terminal deoxynucleotidyl transferase-mediated dUTP-biotin nick end labeling (TUNEL) staining of brain tumors extracted from various groups.

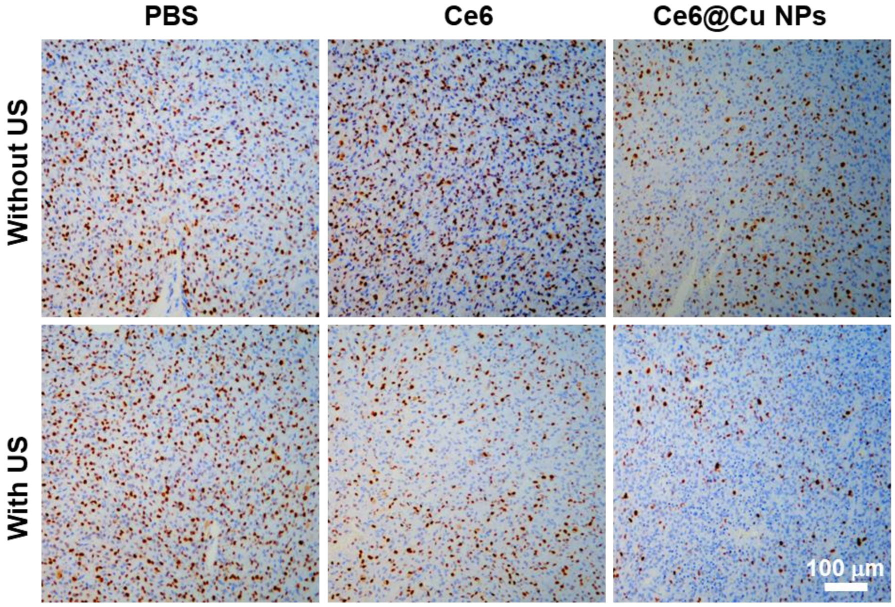

Figure S27. Ki67 staining of brain tumors collected from different groups.

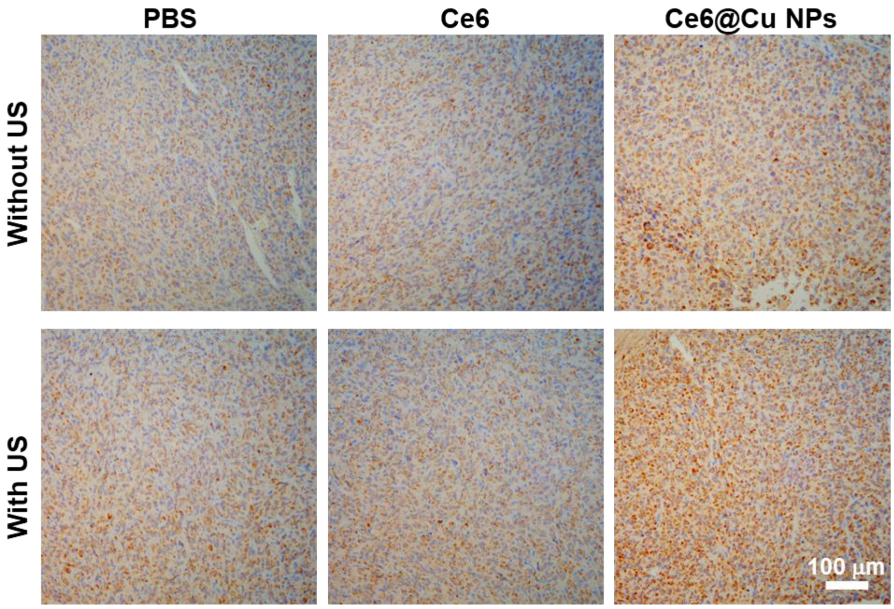

Figure S28. Immunochemical staining of brain tumors for LIAS expressions in different groups.

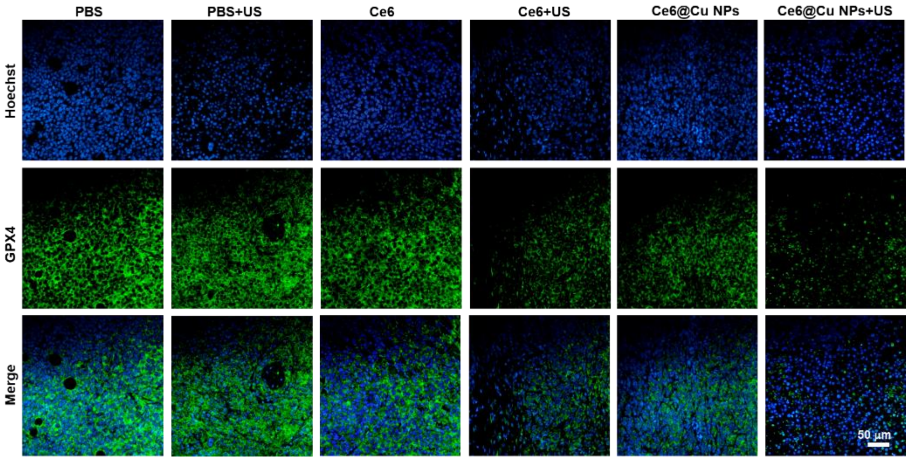

Figure S29. Immunofluorescent staining of brain tumors to assess the expression levels of GPX4 in different groups.

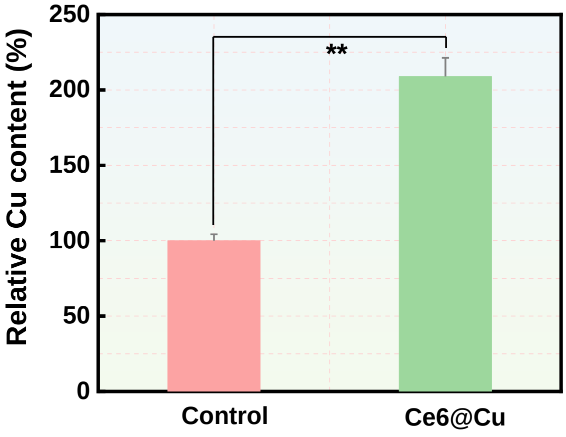

Figure S30. The concentration of copper ion in glioblastoma after different treatments.
